# Rapid cardiac ischemia detection with an epicardial graphene probe

**DOI:** 10.3389/fcvm.2023.1111651

**Published:** 2023-06-22

**Authors:** Grzegorz Suwalski, Marek Galanty, Beata Degórska, Jacek Sterna, Jan Frymus, Mikhal Baranski, Piotr Trębacz, Daniel Janczak, Sandra Lepak-Kuc, Małgorzata Jakubowska

**Affiliations:** ^1^ Department of Cardiac Surgery, Military Institute of Medicine, Warsaw, Poland; ^2^ Department of Small Animal Diseases and Clinic, Faculty of Veterinary Medicine, Warsaw University of Life Sciences, Warsaw, Poland; ^3^ Faculty of Mechatronics, Warsaw University of Technology, Warsaw, Poland

**Keywords:** cardiac ischemia, epicardial electrocardiography, coronary artery bypass grafting, cardiac surgery, myocardial infarction

## Abstract

**Introduction:**

In this study, a new probe was designed to enable electrocardiography of a rotated heart during cardiac surgery when skin electrodes became non-functional. This probe adhered non-invasively to the epicardium and collected the ECG signal independently from the position of the heart. The study compared the accuracy of cardiac ischemia detection between classic skin and epicardial electrodes in an animal model.

**Methods:**

Using six pigs, an open chest model was devised with cardiac ischemia induction by coronary artery ligation in two non-physiologic heart positions. Both the accuracy and the time of detection of electrocardiographic symptoms of acute cardiac ischemia were compared between skin and epicardial methods of signal collection.

**Results:**

Heart rotation to expose either the anterior or the posterior wall resulted in a distortion or loss of the ECG signal collected by skin electrodes after coronary artery ligation, standard skin ECG monitoring did not reveal any ischemia symptoms. Attachment of an epicardial probe on the anterior and posterior walls helped in the recovery of the normal ECG wave. After ligation of the coronary artery, the epicardial probes recorded cardiac ischemia within 40 s.

**Discussion:**

This study highlighted the effectiveness of ECG monitoring with epicardial probes in a rotated heart. It can be concluded that epicardial probes can detect the presence of acute ischemia of a rotated heart when skin ECG monitoring becomes ineffective.

## Introduction

Coronary artery bypass grafting (CABG) is the most common heart surgery performed worldwide. In nearly half of heart patients, CABG is being performed on a beating heart–off-pump. During this surgery, exposure of the target coronary arteries requires a change in the position of the heart; however, this maneuver often leads to a loss of contact between the heart and the adjacent chest structures. As a result, there is a loss of proper electric signal conduction from the heart to the electrocardiographic (ECG) electrodes applied on a patient's skin. Thus, the ECG signal during the performance of off-pump coronary artery bypass (OPCAB) is often lost or distorted, placing restrictions on the monitoring of cardiac ischemia during surgery. In light of the above, and with the objective of restoring optimal ECG monitoring during heart positioning, a new epicardial ECG electrode was designed in this study.

Using an animal beating heart model, this study aimed to compare the two methods of ECG monitoring of a rotated heart: standard monitoring with signal collection by skin electrodes and a new method of monitoring with epicardial signal recording. The comparison included testing the accuracy and rapidity of myocardial ischemia detection and the safety of electrode use.

## Materials and methods

**Figure 1:**
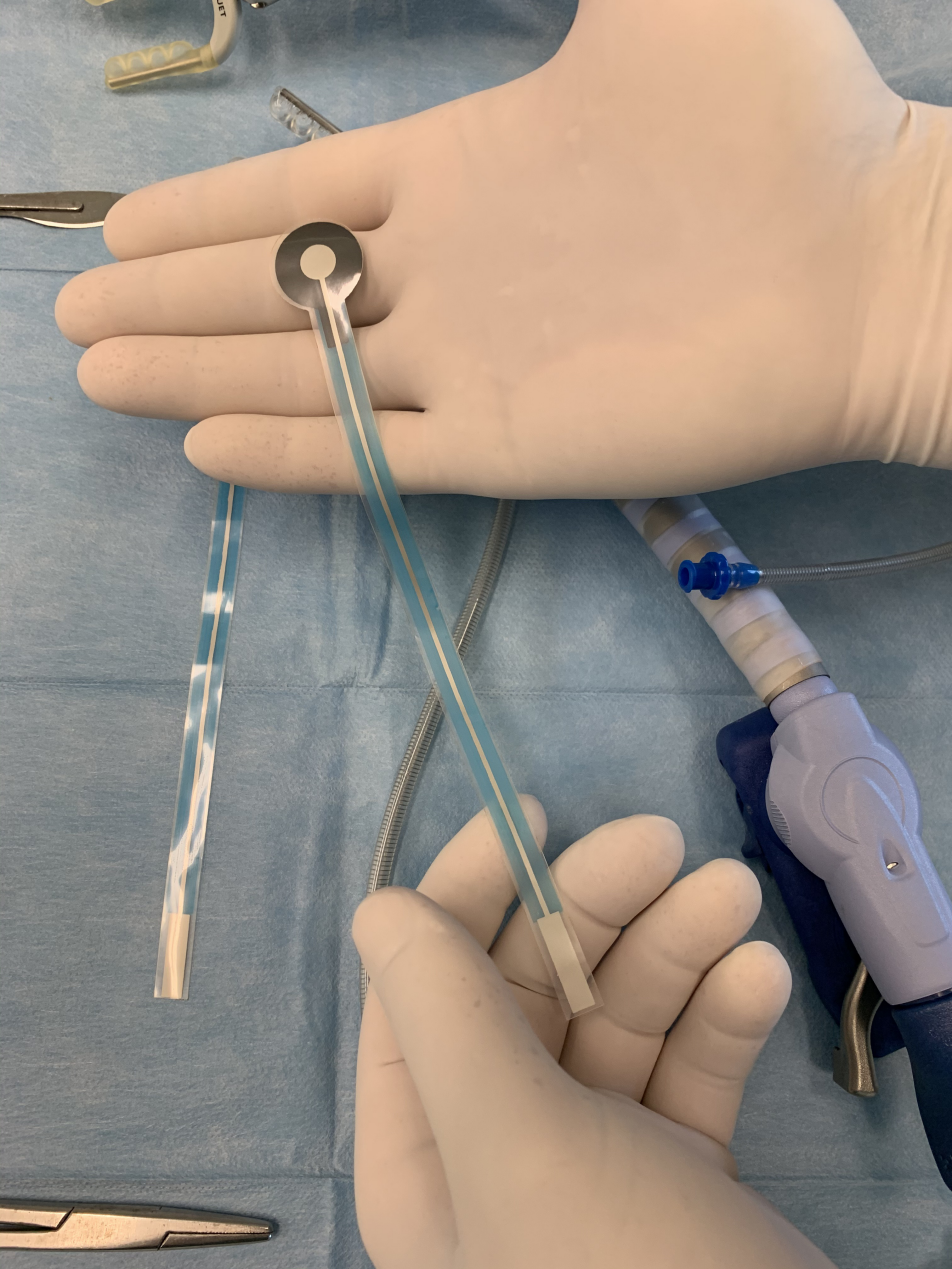
Presentation of a new epicardial probe.

The new epicardial probe was designed as a thin and elastic slice manufactured with screen printing technology (Figure 1) with a circular proximal ending (head) of 2 cm in diameter. The head of the electrode was attached to the heart and the distal part was 19 cm long with a 1- cm-wide branch (tail). The tail was designed to range out of the operative field (open chest) and connect with a dedicated wire. At the head of the probe, an adhesive layer was created to enable non-invasive attachment to the surface of the beating heart and keep the probe in the desired position on all cardiac walls. The adhesion was reinforced with an adhesion promotor (starch), and maintenance of the probe attached to the cardiac wall did not require suturing.

**Figure 2:**
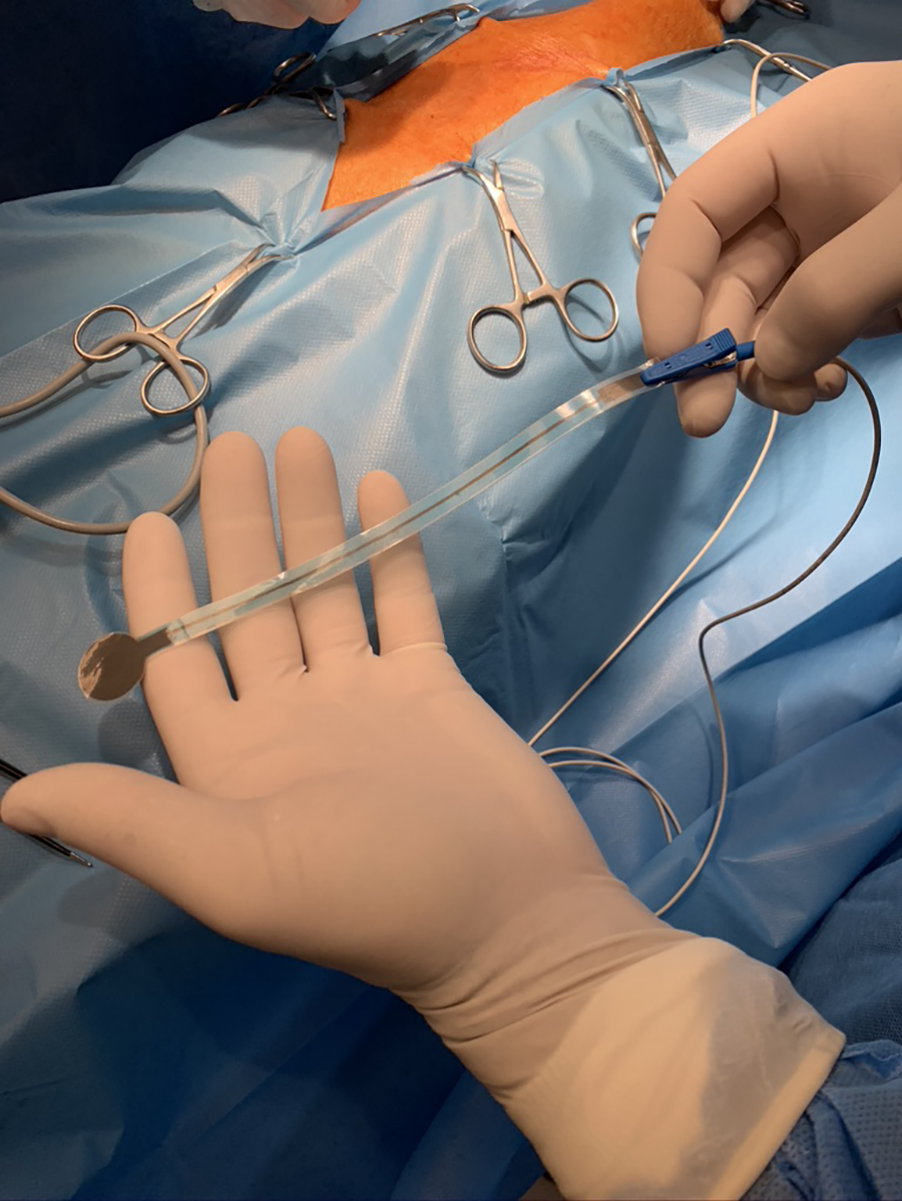
Connection of an epicardial probe with a dedicated wire.

**Figure 3:**
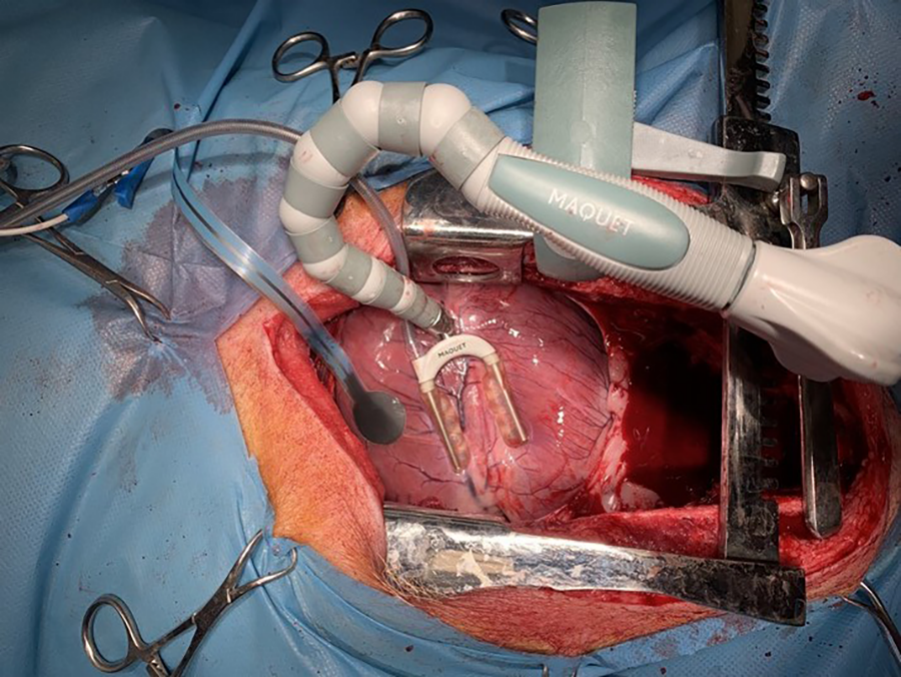
Epicardial probe and heart stabilizer applied on the anterior wall of the animal's heart.

In the electrode head and tail, there was a conduction layer containing graphene, which was isolated all along the tail. The tip of the tail had a connective platform for wire attachment with a standard crocodile clip, and the distal ending of the wire had a standard ECG snap (Figure 2). The transition from skin ECG monitoring was achieved by disconnecting the ECG wire from the skin probe (lead V) and attaching it to a dedicated probe wire. In such an assembly, the epicardial ECG signal was presented on the cardiomonitor as lead V. Using a study protocol approved by a Local Ethics Committee for Animal Experiments, six domestic pigs (three males and three females) weighing 60–70 kg were anesthetized and intubated. Electrocardiography was monitored in standard fashion using five skin leads and a cardiomonitor (EDAN X, Edan Ltd.). The chest was opened by performing a full median sternotomy. Similar to the coronary artery bypass grafting procedure, the heart was rotated to expose the anterior wall with the left anterior descending (LAD) coronary artery and then to expose the posterior wall with the posterior descending artery (PDA). The epicardial probe was then attached on either the anterior or the posterior wall, respectively (Figure 3). The probe was connected as lead V. The range of the ECG curve display (mV) was set for automatic scaling with maximal signal reduction by a factor of 0.125. Induction of cardiac ischemia was performed with the ligation of the LAD artery in three animals and the PDA in three animals. Then, ECG signals collected with standard skin and epicardial electrodes were compared. The presence of acute cardiac ischemia was detected when the elevation of the ST segment in the ECG curve exceeded 0.1 mV. The time from coronary artery ligation to appointed ST segment elevation (0.1 mV) was recorded. After ischemia induction, the probes were kept in one position until the occurrence of severe heart rhythm disturbances (ventricular tachycardia or fibrillation). Then, euthanasia was performed by administering an intravenous pentobarbital injection (13 mg per kg). The safety of the new probe application was assessed by a macroscopic analysis of the epicardial areas following its detachment, and the tissue was observed for tearing, bleeding, hematoma, or left probe debris. All procedures conformed to the guidelines of Directive 2010/63/EU of the European Parliament.

## Results

During the chest opening procedure, skin electrodes showed normal ECG levels with no cardiac ischemia or any heart rhythm disturbances in all animals. In the first three animals, the heart was rotated to expose the LAD artery. During the rotation, the ECG skin signal was distorted in two animals (no diagnostic value) and was completely lost in one. Attachment of an epicardial probe resulted in the recovery of ECG presentation as lead V. The morphology of the epicardial ECG signal was typical for physiologic precordial leads showing no signs of ischemia (no ST segment elevation in all animals). After LAD ligation, the skin ECG revealed either no change (one animal) or an ST segment elevation change of less than 0.1 mV (no ischemia). The epicardially collected ECG showed acute cardiac ischemia with ST segment elevation exceeding 1 mV (Pardee wave) in all three animals at a duration ranging between 15 and 30 s.

**Figure 4:**
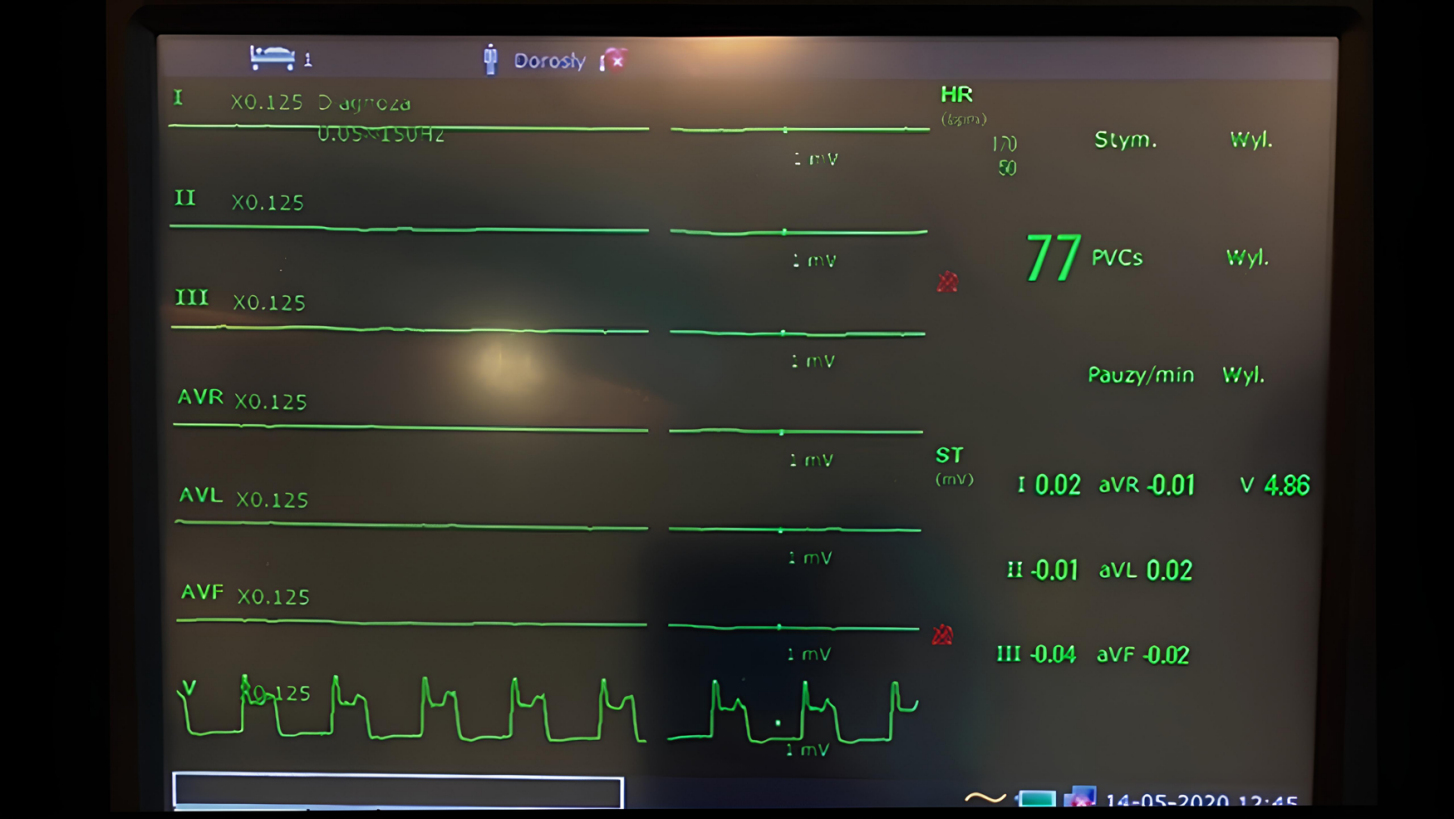
Monitor screen showing myocardial ischemia detected with an epicardial probe (lead V) when the signal from skin electrodes was lost due to heart rotation.

In the remaining three animals, the experiment heart was rotated to expose the PDA. During the rotation, the ECG skin signal was lost in all animals. The epicardial probe was attached to the posterior wall, which resulted in the restoration of the ECG curve in lead V. Also, the morphology of the epicardial ECG signal in this position was typical for a physiologic precordial lead showing no signs of ischemia (no ST segment elevation in all animals). Then, the PDA was ligated. No reaction was recorded in the ECG collected with skin electrodes, since the signal was already lost. The epicardial probe located on the posterior wall revealed ST segment elevations exceeding 1 mV (Pardee wave) in all three animals at a duration ranging between 30 and 40 s (Figure 4).

At the end of the experiments, the probes were detached from the epicardium and no macroscopic changes in tissue, such as bleeding, hematoma formation, or tissue tears, were noticed. In addition, there was no debridement of the electrode left on the epicardial tissue, and all probes remained intact and functional until the completion of the study protocol.

## Discussion

Intraoperative ECG monitoring during the performance of OPCAB has not been widely studied, and it has been shown that hemodynamic instability during OPCAB can be secondary to transient ischemia (1, 2). Nevertheless, in clinical practice, it is observed that heart rotation during OPCAB eliminates the diagnostic value of skin ECG monitoring. In an attempt to solve this problem, a few solutions of intraoperative cardiac ischemia monitoring were tested by some clinicians. One study presented a technology of a heart stabilizer with an integrated ECG monitoring system (3). The feasibility of intraoperative cardiac ischemia monitoring was also examined by using intravascular near-infrared spectroscopy (4); however, the usefulness of the gathered data using this technique has yet to be validated. A few studies analyzed the usefulness of trans-esophageal echocardiography in intraoperative monitoring of heart contractility (5), but this method is limited by the advanced rotation of the heart. However, none of these concepts have been introduced in clinical practice, and therefore, the abovementioned problem remains unsolved.

The introduction of the presented technology may result in several benefits for patients undergoing OPCAB. First, the provision of continuous information on cardiac ischemia during the performance of coronary anastomosis may reduce the pressure of time for surgeons. This may result in an anastomosis of high quality. Some data showed lower anastomosis quality in OPCAB when compared with the arrested heart technique (6).

Second, epicardial ECG monitoring may support the decision-making process in the positioning of the heart for exposure of the target coronary arteries. An inappropriate position may result in ischemia induction before coronary artery ligation, hemodynamic collapse, and poor coronary bypass performance, leading to incomplete revascularization affecting long-term results (7).

Third, immediate ischemia diagnosis provides immediate actions, such as intracoronary shunt placement, repositioning of the heart, arterial pressure management, or even conversion to an on-pump technique before hemodynamic collapse.

A review published by the ATLANTIC group showed that intraoperative cardiac ischemia was a reason for emergency conversion to on-pump surgery in 17% of patients with this condition. Moreover, this review revealed that in 18% of patients requiring emergency conversion to the on-pump technique, intraoperative myocardial infraction was diagnosed (8). The consequences of such conversion are often catastrophic. Borde et al. demonstrated that emergency conversion increases the risk of death by 16 times (1.7% of deaths in the non-conversion group vs. 16.4% in the conversion group; *p* = 0.0001) (9).

Epicardial electrocardiography will help in OPCAB technique expansion, which is a less invasive method than the classic on-pump technique with the use of cardio-pulmonary bypass (heart-lung machine). It should be noted that both short- and long-term results of OPCAB are related to the number of procedures performed in patients (10); the more patients are treated with the OPCAB technique, the better the results will be. Thus, any device making the OPCAB technique easier and safer may influence the final results.

The new epicardial device designed in this study has several other applications in cardiac surgery and may also be used as a temporary external pacing wire, or for electrical mapping of the pulmonary veins while monitoring the entry and exit block after pulmonary vein isolation. Since the signal collected by the probe is strong and clear, it may support an intraoperative assessment of the vitality of the cardiac wall regions.

The limitation of this study is its small sample size of an animal model, and future clinical studies will evaluate whether any benefits can be had from the introduction of epicardial ECG monitoring.

In conclusion, this study highlighted the effectiveness of ECG monitoring with epicardial probes in a rotated heart. Epicardial probes can detect the presence of acute ischemia of a rotated heart when skin ECG monitoring becomes ineffective. Epicardial ECG monitoring can potentially replace standard skin ECG monitoring during the performance of beating heart surgery.

## Data Availability

The raw data supporting the conclusions of this article will be made available by the authors without undue reservation.
