# Supplementary figures and images for: Rapid cardiac ischemia detection with an epicardial graphene probe
Source: Front Cardiovasc Med. 2023 Jun 22;10:1111651. doi: 10.3389/fcvm.2023.1111651 (PMC10323424; doi:10.3389/fcvm.2023.1111651)

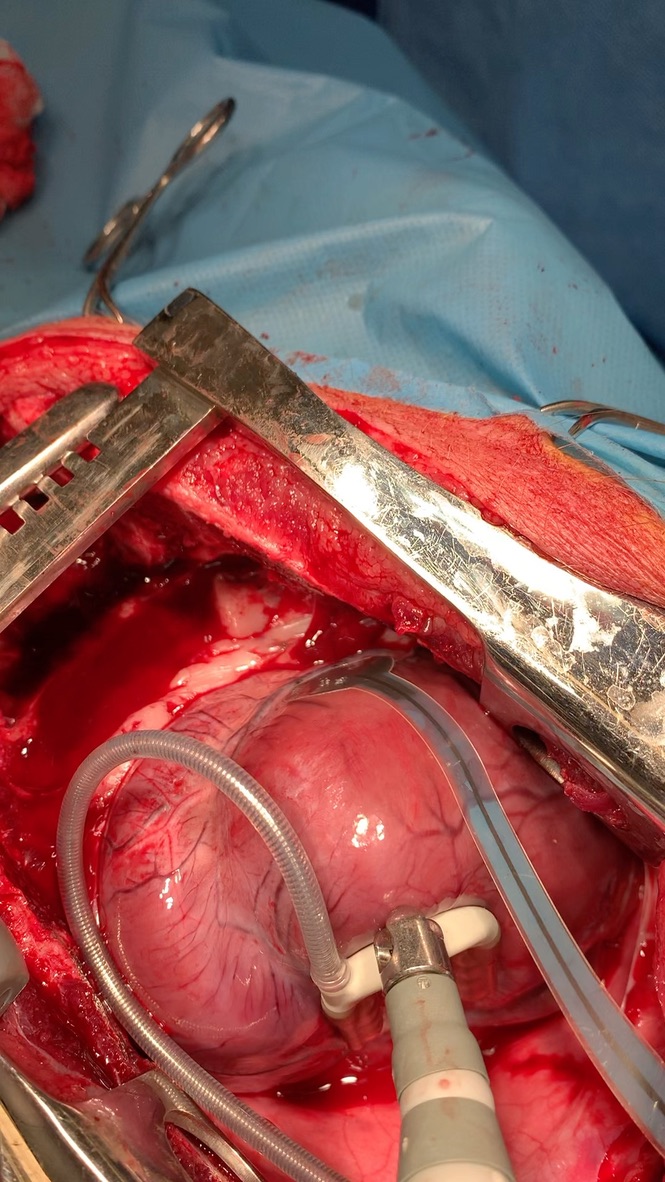

Supplement: Supplementary file 1 [file Image1.jpeg]

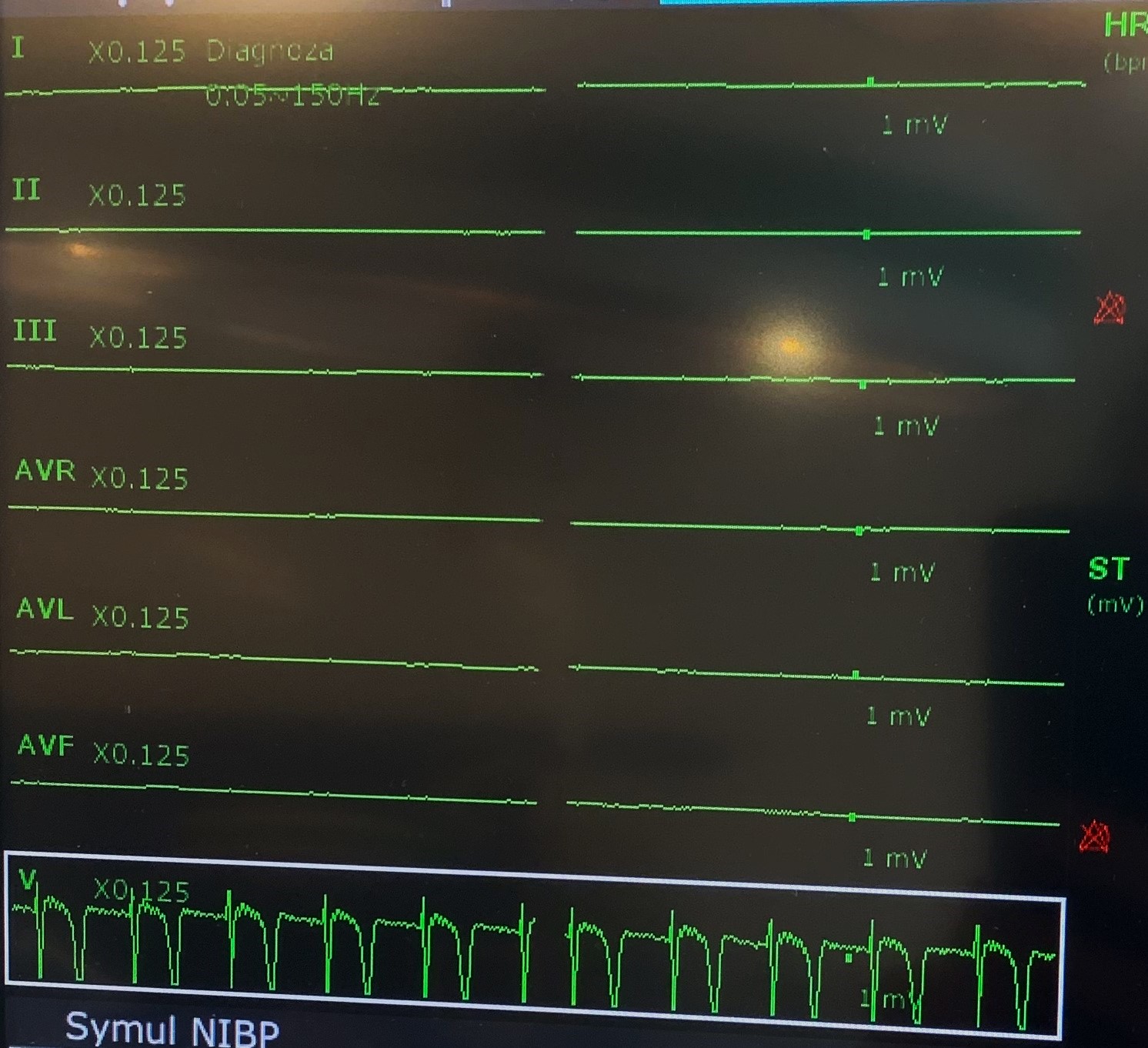

Supplement: Supplementary file 2 [file Image2.jpeg]
